# Hydrodynamic Compatibility of Hyaluronic Acid and Tamarind Seed Polysaccharide as Ocular Mucin Supplements

**DOI:** 10.3390/polym12102272

**Published:** 2020-10-02

**Authors:** Taewoo Chun, Thomas MacCalman, Vlad Dinu, Sara Ottino, Mary K. Phillips-Jones, Stephen E. Harding

**Affiliations:** 1 National Centre for Macromolecular Hydrodynamics (NCMH), School of Biosciences, University of Nottingham, Sutton Bonington LE12 5RD, UK; stxtc16@exmail.nottingham.ac.uk (T.C.); stxtm22@exmail.nottingham.ac.uk (T.M.); sbavd@exmail.nottingham.ac.uk (V.D.); 2 Farmigea S.P.A, Via G.B. Oliva, 6/8 - 56121 Pisa, Italy; s.ottino@farmigea.it; 3 Cultural History Museum, University of Oslo, Postboks 6762, St. Olavs plass, 0130 Oslo, Norway

**Keywords:** molar mass, heterogeneity and conformation, analytical ultracentrifugation, light scattering, viscometry

## Abstract

Hyaluronic acid (HA) has been commonly used in eyedrop formulations due to its viscous lubricating properties even at low concentration, acting as a supplement for ocular mucin (principally MUC5AC) which diminishes with aging in a condition known as *Keratoconjunctivitis sicca* or “dry eye”. A difficulty has been its short residence time on ocular surfaces due to ocular clearance mechanisms which remove the polysaccharide almost immediately. To prolong its retention time, tamarind seed gum polysaccharide (TSP) is mixed as a helper biopolymer with HA. Here we look at the hydrodynamic characteristics of HA and TSP (weight average molar mass *M_w_* and viscosity $\left[\eta \right]$) and then explore the compatibility of these polymers, including the possibility of potentially harmful aggregation effects. The research is based on a novel combination of three methods: sedimentation velocity in the analytical ultracentrifuge (SV-AUC), size-exclusion chromatography coupled to multiangle light scattering (SEC-MALS) and capillary viscometry. HA and TSP were found to have $M_{w}=(680\pm 30$) kg/mol and (830±30) kg/mol respectively, and $\left[\eta \right]=\left(1475\pm 30\right)\;\mathrm{ml}/g$ and $\left(675\pm 20\right)$ ml/g, respectively. The structure of HA ranges from a rodlike molecule at lower molar masses changing to a random coil for *M*_w_ > 800 kg/mol, based on the Mark–Houwink–Kuhn–Sakurada (MHKS) coefficient. TSP, by contrast, is a random coil across the range of molar masses. For the mixed HA-TSP systems, SEC-MALS indicates a weak interaction. However, sedimentation coefficient (*s*) distributions obtained from SV-AUC measurements together with intrinsic viscosity demonstrated no evidence of any significant aggregation phenomenon, reassuring in terms of eye-drop formulation technology involving these substances.

## 1. Introduction

As people age, their production of ocular mucin containing lacrymal fluid—with its natural protective and lubricating properties for the surface of the eye—diminishes, a term known medically as *Keratoconjunctivitis sicca* or “dry eye”. Solutions of polysaccharide in artificial tear drop formulations are popularly used to consolidate the mucin (of primarily type MUC5AC, also MUC2) to remove these symptoms. A problem with such formulations is how to prolong their residence time on the eye surface. The main reason for this problem is that ocular protective mechanisms (involving blinking, both basal and reflex lachrymation, and a drainage through nasolacrimal ducts) quickly eliminates these eye drops from the precorneal region, where such drugs are absorbed and work [1]. As a result, and depending on the severity, this can lead to the need for repeated administration [2]. Therefore, various studies have investigated novel preparations to overcome such disadvantages of currently available formulations. For example, based on the viscosity enhancing effects of gelatin, the grafting of thermoresponsive polymer segments onto proteinaceous networks has led to promising results [3] and, more recently, the lectin *Helix pomatia* agglutinin has been considered as an ocular mucoadhesive component [4]. The properties of a promising new glutathione-dependent polymeric hydrogel with good eye drop mucoadhesive properties has also been explored [5].

**Figure 1:**
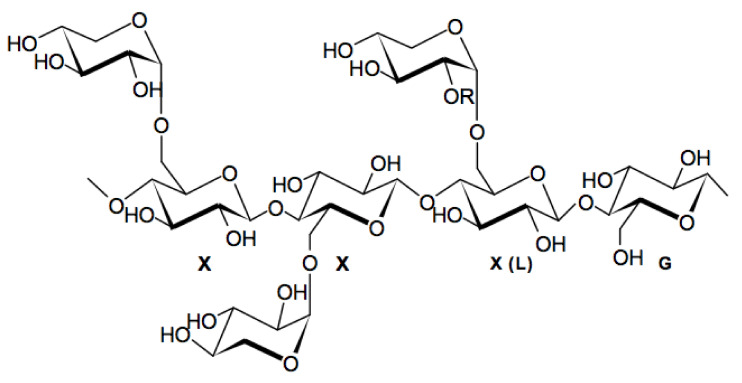
Chemical structure of tamarind seed xyloglucan; motif XXXG or β

Another highly significant development has been the combination of hyaluronic acid (HA) and tamarind seed gum polysaccharide (TSP), whose medical benefits were first reported by Barabino et al. [6]. Of the two components, both natural polysaccharides, HA is a linear polyanionic molecule chemically grouped in a glycosaminoglycan and comprises the repeating dimer {→4)-β-D-glucopyranosyluronic acid-(1→3)-*N*-acetyl-2-amino-2-deoxy-β-d-glycopyranosyl-(1→} [7,8]. When it comes to the current market situation, it has been reported [2] that 0.1–0.5% (*w/v*) HA solutions are available commercially as either active or inactive ingredients to supplement ocular fluid (see also refs [9] and [10]). Hammer and Burch [11] have suggested that 0.17% HA showed superior protective effects as a coating on the eye compared to the highly viscous, more concentrated applications (1%, equivalent to 10 mg/mL) which transmit excessive shear force to endothelial cells. An alternative approach, which can also help with product stability issues is to use HA in a binary mixture with another polysaccharide. The second polysaccharide, TSP (Figure 1) is a nonionic, neutral and branched xyloglycan, which is comprised of a cellulose-like backbone, partially replaced at the O-6 position of its glucopyranosyl units with α-d-xylopyranose [6,12].

A comparative study by Rolando and Valente [13] has indicated that both 0.5% and 1% solutions of TSP are comparable to 0.2% HA in terms of removing the symptoms of dry eye syndrome. Later, Barabino et al. [6] published their results with mixtures of HA and TSP, showing that this combination is effective in fixing the tear film on the cornea and repairing the endothelial damage in dry eye patients. One principal reason for these outcomes is that the structural similarity of TSP to transmembrane mucins (MUC1) on the eye surface could lead to its longer retention time [13].

-D-galactopyranose (motif XXLG). Adapted from [14].

In addition, recent evidence using nuclear magnetic resonance spectroscopy (NMR)—a powerful method for investigating macromolecular-ligand interactions [15]—has suggested that there is an interaction between these two polysaccharides [16]. We now seek to reinforce those observations by exploring the hydrodynamic compatibility and stability of these mixtures. Specifically we examine key hydrodynamic parameters such as the molar mass and intrinsic viscosity by size exclusion chromatography coupled to multiangle (laser) light scattering SEC-MALS and capillary viscometry, together with sedimentation velocity in the analytical ultracentrifuge, SV-AUC to assess the heterogeneity and interaction strength. The matrix-free method of analytical ultracentrifugation—with its huge dynamic range (molar masses from 10^2^–10^8^ g/mol) is a key or “gold standard” method used to assess the molecular integrity of other biotherapeutic systems such as monoclonal antibodies (in terms of disassembly, denaturation or aggregation effects) and this is the first time that this method has been used to assess a dry-eye formulation. It has both a high separation and analysis ability without the need of columns or membranes [17,18]. We assess the change in the intrinsic viscosity with molar mass in order to estimate conformations of HA and TSP by SEC-MALS and viscometry, assess the state of self-association/aggregation of the individual components and for interaction/aggregation phenomena of the mixtures using SEC-MALS and SV-AUC, and finally investigate the stability of HA, TSP, and their mixtures.

Although polysaccharides such as cellulose acetate [19], proteins [20] and other natural polymers [21] are commonly used singly in biomedical applications, examples of the combined use of natural polymers for medicinal purposes are not so frequent but are increasing in importance. The HA-TSP system has provided a further good example [1,6,16,22], now reinforced by the present analytical ultracentrifuge-based study.

## 2. Materials and Methods

### 2.1. Materials

Hyaluronic acid and tamarind seed polysaccharide were supplied by Farmigea S.p.A., Pisa, Italy. HA or TSP samples were dissolved in a phosphate-chloride buffered saline solution (PBS, or “Paley buffer”) at pH ~6.8 adjusted to an ionic strength of I = 0.1 mol/L by the addition of NaCl [23].

Stock solutions of HA and TSP were prepared by stirring gently for 30 min followed by overnight dialysis at room temperature against a two-litre volume of PBS. The concentration, *c* (g/mL) of the stock solution (either HA or TSP) was then measured using a differential refractometer (Atago DD7, Tokyo, Japan) set to zero with PBS, and using a refractive increment d*n*/d*c* of 0.167 mL/g for HA, and 0.152 mL/g for TSP [24]. HA/TSP was prepared by adding equal volumes with various concentrations, resulting in a range of ratios (HA:TSP = 1:3, 1:1 and 3:1) and final concentrations (4.0 mg/mL, 2.0 mg/mL and 1.0 mg/mL commensurate with concentrations used in formulations, and with materials remaining in solution (on the premise there are no significant irreversible aggregation/ complex formation interactions).

### 2.2. Sedimentation Velocity in the Analytical Ultracentrifuge

Sedimentation coefficients and sedimentation coefficient distributions were determined using the optimal XL-I analytical ultracentrifuge (Beckman Instruments, Palo Alto, CA, USA) with Rayleigh interference optics. Reference solvent or dialysate (420 μL) and HA, TSP or HA/TSP samples (400 μL) with different concentrations were injected into channels of 12 mm, double-sectored cells with sapphire windows. Then these cells were loaded into an eight-hole rotor and centrifuged at a rotor speed of 45,000 rpm at a temperature of 20.0 °C for a run time of ~24 h. The data was analysed using the SEDFIT algorithm [25], which gives the sedimentation coefficient distribution, $g\left(s\right)$ versus $s_{T,b}$, where s is the sedimentation coefficient, at temperature *T* and in buffer *b*. The s value was then corrected to standard solvent conditions (density and viscosity of water at 20.0 °C) to produce $s_{20,w}$ using the equation [26]:

(1) $$s_{20,w}=\left\{\left(1-\bar{v}\rho_{20,w}\right)/\left(1-\bar{v}\rho_{T,b}\right)\right\}\left\{\eta_{T,b}/\eta_{20,w}\right\}\cdot s_{T,b}$$

where $\bar{v}$ is the partial specific volume of each sample. To eliminate the effect of nonideality, the Gralén equation was used for the extrapolation [27]:

(2) $$\frac{1}{s_{20,w}}=\frac{1}{s_{20,w}^{0}}\left(1+k_{s}c\right)$$

where $k_{s}$ is the Gralén coefficient (mL/g).

### 2.3. Size Exclusion Chromatography Coupled to Multiangle Laser Light Scattering (SEC-MALS)

Weight average molar masses (*M*_w_) of HA, TSP and HA/TSP were estimated by SEC-MALS [7,14,28,29]. The solvent/ buffer was pumped at a steady flow rate of 0.5 mL/min through a column (Shodex LB-805 and), which was protected by a guard column (Shodex LB-G6B), coupled on-line to MALS (Dawn Heleos-11), a differential pressure viscometer (ViscoStar-11) and refractive index (Optilab rEX) detectors (Wyatt Technology, Santa Barbara, CA, USA). After being filtered through a 0.2 μm syringe filter (Whatman, Maidstone, England), the solutions of the HA, TSP, and HA/TSP sample prepared at a concentration of 1.24 mg/mL, 0.68 mg/mL, and 1.0 mg/mL (HA:TSP = 1:1) respectively, were injected into the size exclusion system using the Spark-Holland Marathon Basic autosampler to dilute on the column. ASTRA^TM^ (Version 6.2) software (Wyatt Technology, Santa Barbara, CA, USA) was used to analyse the data. Apparent weight average molar mass (*M*_w,app_) was calculated by using a linear fit to the Zimm model [30]:

(3) $$\frac{Kc}{R_{\theta }}=\left(\frac{1}{M_{w}}+2Bc\right)[1+\frac{16\pi^{2}\bar{R_{g}^{2}}}{3\lambda^{2}}\sin^{2}\frac{\theta }{2}]$$

where *K* is an experimental constant dependent on the wavelength of the light and the refractive index increment of the polysaccharide, $R_{\theta }$ is the Rayleigh ratio used to determine the ratio of the integrity of light scattered by a macromolecule at an angle θ to that of the incident radiation, *B* is the second virial coefficient (mL·mol·g^−2^), $R_{g}$ is the radius of gyration (cm) [31].

*M*_w,app_ is obtained from the intercept [29]. We make the reasonable assumption that correction for thermodynamic nonideality was assumed to be unnecessary due to the high dilutions on the SEC columns (see, for example ref [32]) and hence *M*_w_ the “ideal” molar mass ~*M*_w,app_.

### 2.4. Capillary Viscometry

HA solutions ranging from 0.08 to 0.62 mg/mL, TSP solutions (from 0.03 to 1.91 mg/mL), and HA/TSP solutions (1 to 4 mg/mL) were analysed using a semiautomated viscosity measuring system (AVS 400, Schott Geräte, Hofheim, Germany) at a temperature of 20.00 °C in a 2 mL Ostwald viscometer (Table 1). Considering the fact that, because of low concentration (<2 mg/mL), a density correction is considered to be redundant [14], the relative viscosity, $\eta_{\mathrm{rel}}$, was estimated to be the ratio of the flow time of the solution t (sec) to that of solvent $t_{0}$:

(4) $$\eta_{\mathrm{rel}}\approx \frac{t}{t_{0}}=\eta_{\mathrm{sp}}+1$$

where $\eta_{\mathrm{sp}}$ is the specific viscosity [33]. Then the reduced specific viscosity, $\eta_{\mathrm{red}}$, (mL/g) and the inherent viscosity, $\eta_{\mathrm{inh}}$, (mL/g) were obtained from:

(5) $$\eta_{\mathrm{red}}=\left(\eta_{\mathrm{rel}}-1\right)/c$$

(6) $$\eta_{\mathrm{inh}}=\left(\ln\;\eta_{\mathrm{rel}}\right)/c$$

where c (g/mL) is the solute concentration [33].

These $\eta_{\mathrm{red}}$ and $\eta_{\mathrm{inh}}$ can be extrapolated to zero concentration in order to eliminate nonideality effect, leading to the intrinsic viscosity $\left[\eta \right]$:

(7) $$\eta_{\mathrm{red}}=\left[\eta \right]\;\left(1+K_{H}\cdot \left[\eta \right]\cdot c\right)$$

(8) $$\eta_{\mathrm{inh}}=\left[\eta \right]\;\left(1-K_{K}\cdot \left[\eta \right]\cdot c\right)$$

where $K_{H}$ is the Huggins constant [34] and $K_{K}$ is the Kraemer constant [35]. Additionally, a combination of Equations (7) and (8) was used [36]:

(9) $$\left[\eta \right]≃\frac{1}{c}\cdot {\left[2\eta_{\mathrm{sp}}-2\ln\left(\eta_{\mathrm{rel}}\right)\right]}^{1/2}$$

## 3. Results and Discussion

### 3.1. Comparison of Hydrodynamic Properties of HA and TSP of the Preparations

**Figure 2:**
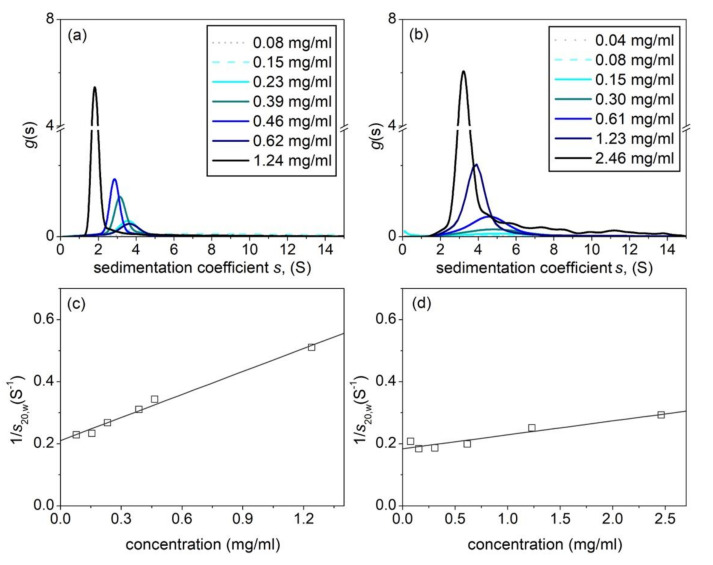
Sedimentation coefficient distributions. (**a**) Hyaluronic acid, HA. (**b**) Tamarind seed polysaccharide, TSP. (**c**) Corresponding concentration extrapolation to zero concentration to eliminate nonideality effects for HA (**d**) Corresponding plot for TSP. Solution pH = 6.8, I = 0.1, temperature = 20.0 °C.

**Figure 3:**
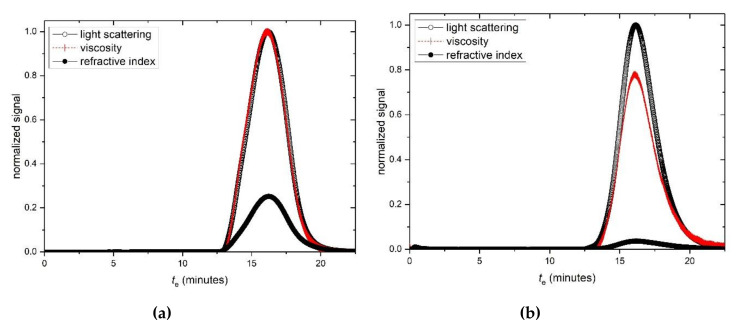

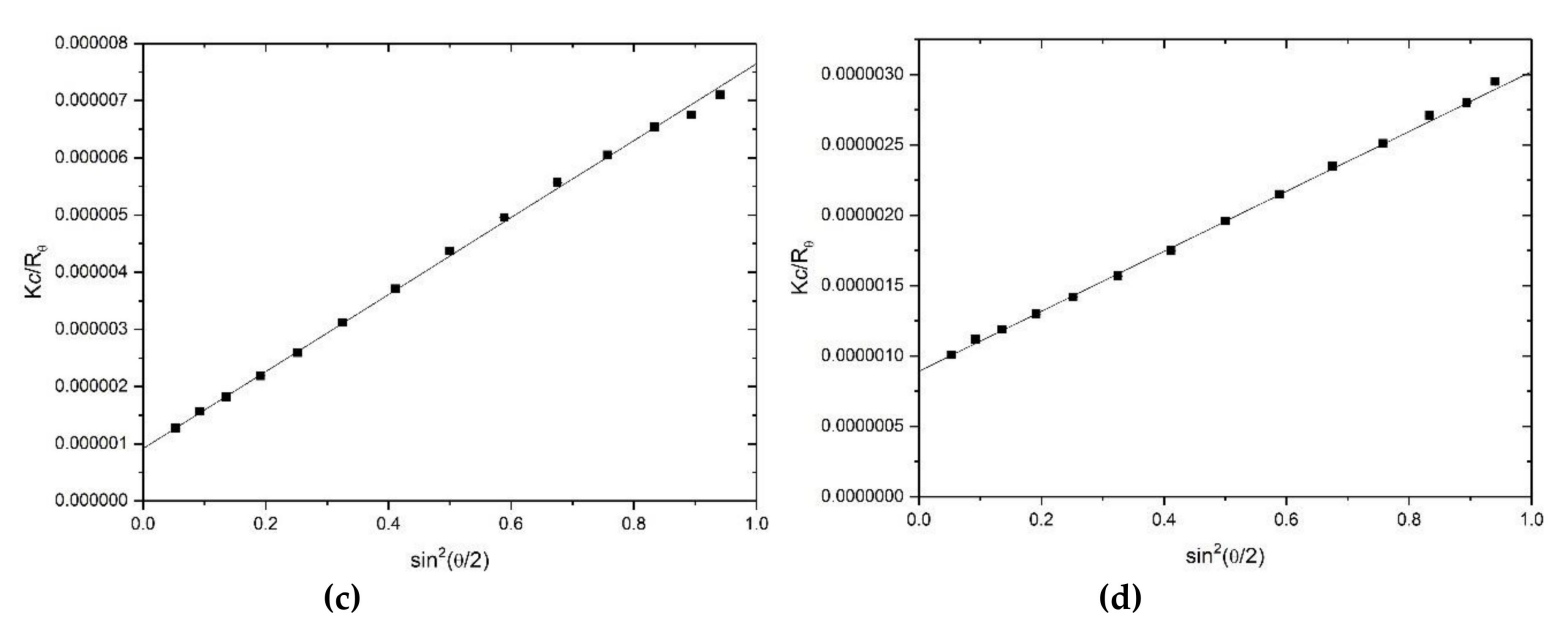
Size exclusion chromatography—multiangle light scattering (SEC-MALS) elution profiles for (**a**) hyaluronic acid, HA and (**b**) tamarind seed polysaccharide, TSP. Solid black circles: light scattering signal at a scattering angle θ = 90°. Open circles: concentration (refractive index) signal; dashed line: differential pressure (viscosity) signal; *t*_e_ = elution time. (**c**) Zimm fit of K*c*/R_θ_ (see text for definitions) vs sin^2^(θ/2) for HA, at a single elution time *t*_e_. (**d**) Corresponding plot for TSP. Solution pH = 6.8, I = 0.1, temperature = 20.0 °C.

Table 1 summarizes the hydrodynamic properties of the hyaluronic acid and tamarind seed gum preparations (supplied by Farmigea AG) in the phosphate-chloride buffer. Sedimentation velocity (Figure 2a,b) and the elution profiles from SEC MALS (Figure 3a,b) show unimodal behaviour for both HA and TSP. Extrapolation of the sedimentation coefficients to zero concentration (Figure 2c,d) yield $s_{20,w}$values of (4.7 ± 0.2)S and (5.4 ± 0.2)S, with concentration dependence or Gralén “*k*_s_” parameter values, respectively of (1171 ± 20) ml/g and (240 ± 30) ml/g. The lower value for *k*_s_ for TSP is commensurate with the presence of weak self-associative effects we previously reported [14] which has an opposing effect on the concentration dependence to hydrodynamic nonideality.

SEC-MALS elution profiles for HA and TSP respectively are given in Figure 3a,b, and corresponding typical Zimm extrapolations for specific elution volumes or times are given in Figure 3c,d. Weight average molar masses *M*_w_ of (680 ± 30) kg/mol and (830 ± 30) kg/mol for the HA and TSP preparations are obtained, with low polydispersities commensurate for an optical formulation.

**Figure 4:**
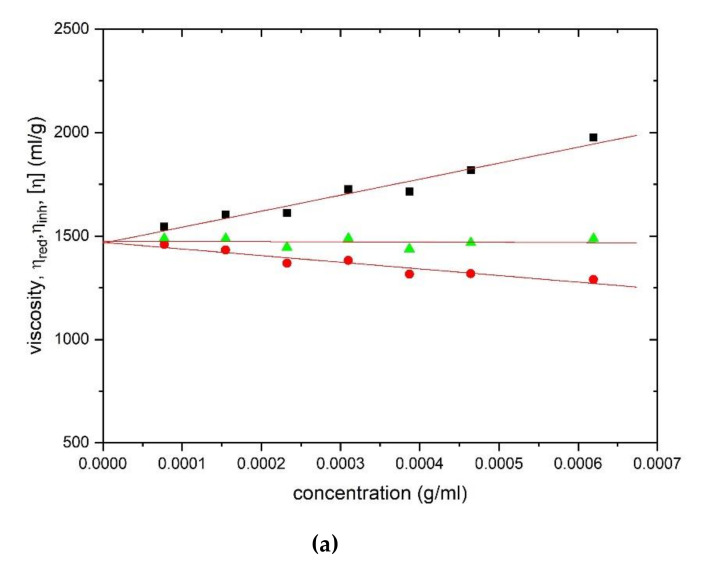

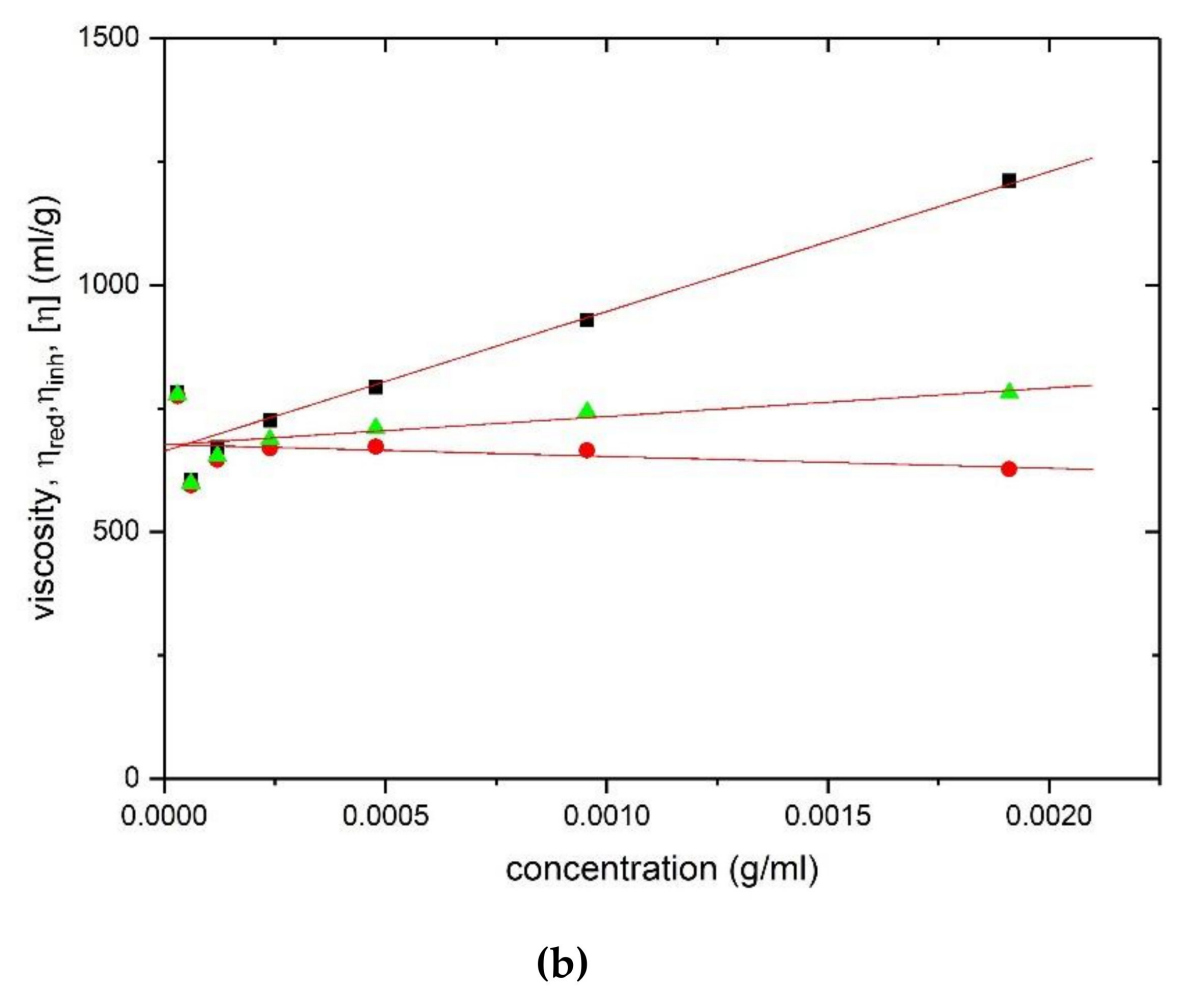
Evaluation of the intrinsic viscosity [η] from Ostwald viscometry: (**a**) hyaluronic acid and (**b**) tamarind seed polysaccharide. Squares: Huggins’ extrapolation; triangles: Solomon–Ciuta; circles: Kraemer extrapolation. Solution pH = 6.8, I = 0.1, temperature = (20.00 ± 0.05) °C.

Corresponding viscosity plots obtained with the Ostwald capillary viscometer are shown in Figure 4 for HA (Figure 4a) and TSP (Figure 4b) respectively, yielding values of (1475 ± 30) ml/g and (675 ± 20) ml/g.

Values for the molar mass and intrinsic viscosity for the Farmigea hyaluronic acid preparations (Table 1) are somewhat lower than we analysed previously from a different source [7,8]. By contrast the tamarind seed gum preparations gave similar results to what we have found before [14,29]. The differential pressure Viscostar (Wyatt Technology, Santa Barbara USA) viscometer attached on-line to SEC allows relative viscosities η_rel_(*t_e_*) to be recorded as a function of elution volume (or elution time *t*_e_), and also the corresponding molar masses *M*_w_(*t_e_*).

Since the concentration is known also for each value of *t_e_* from the refractive index detection, this enables an estimate for [η](*t_e_*) to be obtained from equation (9). Mark–Houwink–Kuhn–Sakurada (MHKS) plots of log [η](*t_e_*) vs log *M*_w_(*t_e_*) for HA and TSP respectively are given in (Figure 4a,b), enabling evaluation of the MHKS conformation parameter *a*:

[η](*t*_e_) = *M*_w_(*t*_e_)*^a^* (10)

**Figure 5:**
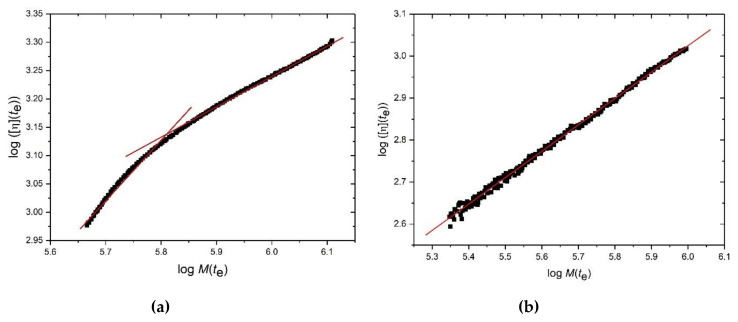
Mark–Houwink–Kuhn–Sakurada plots of log intrinsic viscosity vs log molecular weight *M*(*t*_e_) at corresponding elution times *t*_e_: (**a**) hyaluronic acid and (**b**) tamarind seed gum. Solution pH = 6.8, I = 0.1, temperature = (20.0 ± 0.1) °C.

From Figure 5a (HA) a value of *a* of 1.1 is obtained, corresponding to a stiff extended conformation, although >*M* = 800 kg/mol a lower value is obtained (*a* ~0.56) corresponding to a flexible random coil conformation. Figure 5b we obtain *a* = 0.63 for TSP, also corresponding to a flexible random coil.

### 3.2. Hydrodynamic Behaviour of Mixtures of HA and TSP

**Figure 6:**
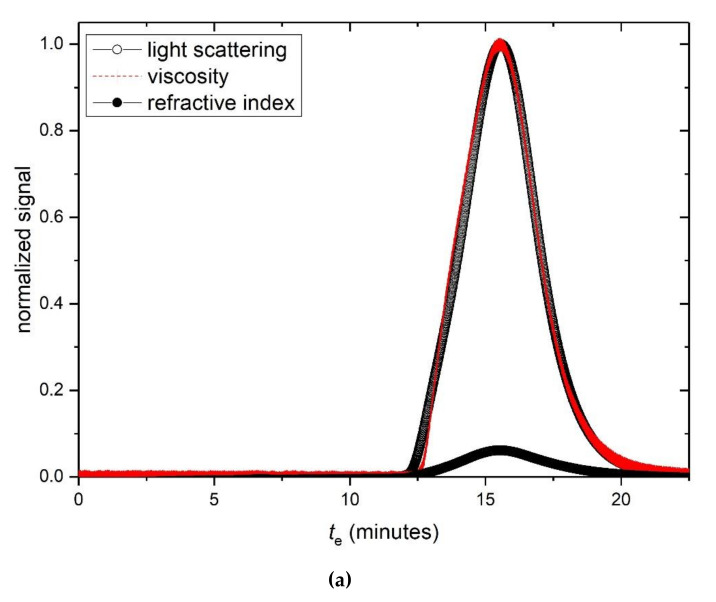

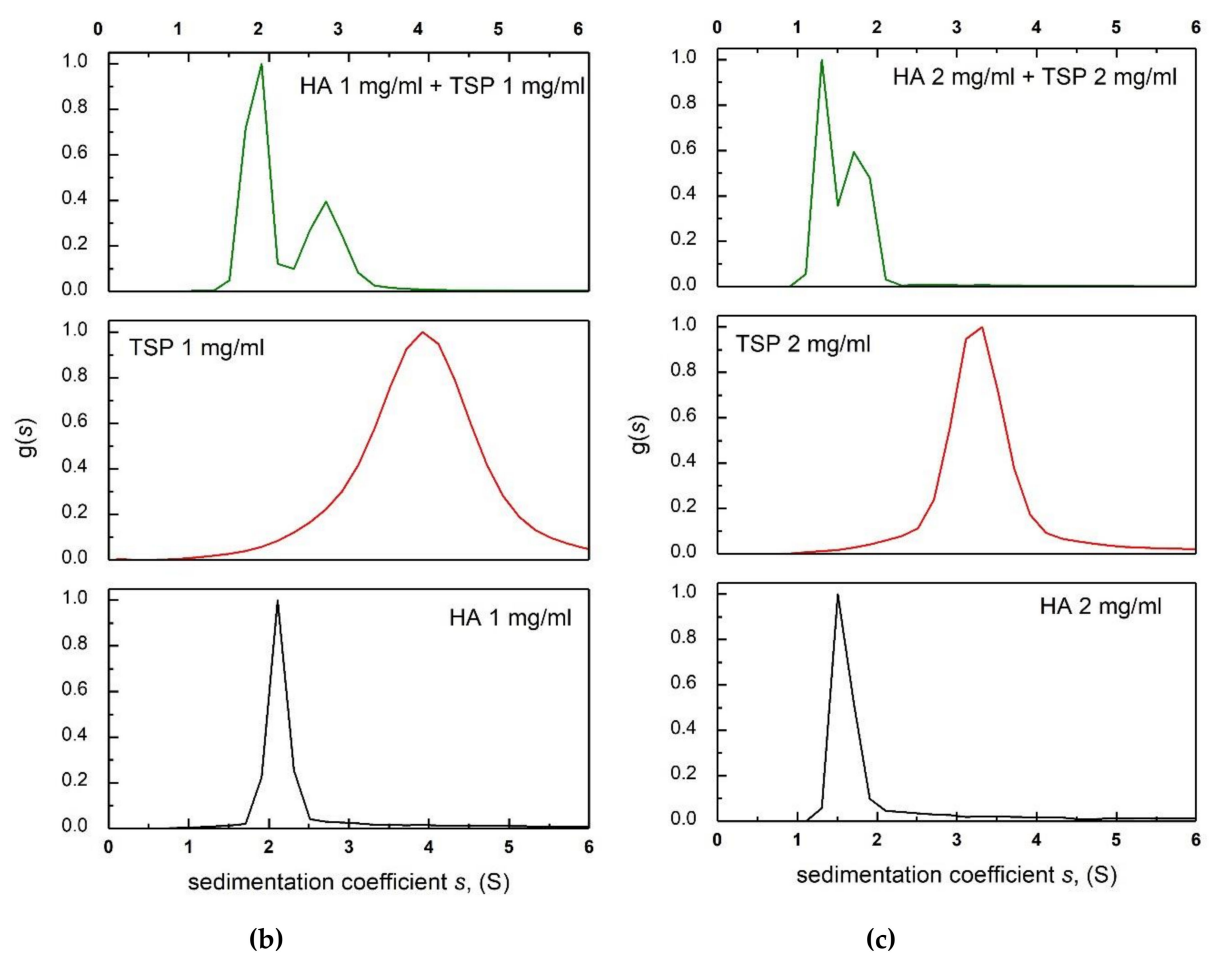
(**a**) Elution profiles for a 1:1 mixture of HA to TSP in 0.1M, pH 6.8 PBS buffer. Solid circles—light scattering 90° signal. Open circles—concentration (refractive index) signal. Dashed line—relative viscosity signal. Concentration of HSA = 1 mg/mL, and TSP= 1 mg/mL. (**b**) Sedimentation coefficient distributions for HA (black), TSP (red) and HA-TSP mixture (green). Concentrations of HA, TSP 1 mg/mL. (**c**) As (**b**) but concentrations of HA, TSP = 2.0 mg/mL.

Figure 6a shows the SEC-MALS profile for a mixture of HA with TSP. One can see a slight shift to lower elution times (higher molar masses) relative to HA and TSP by themselves (Figure 3a,b) with a shoulder at lower elution time suggestive of some degree of complexation or weak interaction. Analysis using ASTRA software shows agreement in the measured *M*_w_ (= 720 ± 30) kg/mol compared with the predicted value of 755 kg/mol.

These observations are reinforced by the results from intrinsic viscosity. Table 2 shows the comparison of the theoretical intrinsic viscosity calculated from the ratio of HA to TSP in each mixture with varying ratios (the HA:TSP ratios = 3:1, 1:1 and 3:1), and the actual intrinsic viscosity obtained experimentally from the capillary viscometry. It is clear that there is no significant increase (HA:TSP = 1:1 and 1:3).

## 4. Conclusions

The present findings of this hydrodynamic study using mixtures of hyaluronic acid and tamarind seed polysaccharides implies there is no incompatibility in solutions of the two, at concentrations used in popular formulations to help supplement reduced lacrymal fluid/ ocular mucin in an aging population. Earlier observations using NMR spectroscopy [1] have demonstrated a synergistic interaction between the two polysaccharides. The present study complements those observations by showing that no large aggregate or supramolecular formation was evident, reassuring for formulations involving the two polymers.

## Figures and Tables

**Table 1:** Hydrodynamic properties of hyaluronic acid (HA) and tamarind seed gum polysaccharide (TSP) in PBS “Paley” buffer.

Sample	HA	TSP
[*η*] (ml/g)	1475±30	675±20
$s_{20,w}^{0}\;\left(S\right)$	4.7±0.2	5.4±0.2
a	1.1*	0.63
$\;10^{-3}{\times M}_{w}\;\left(g/\mathrm{mol}\right)$	680±30	830±30
$\;10^{-3}{\times M}_{z}\;\left(g/\mathrm{mol}\right)$	730	940
$\;10^{-3}{\times M}_{n}\;\left(g/\mathrm{mol}\right)$	640	680
$M_{z}/M_{w}$	1.1	1.1
$M_{w}/M_{n}$	1.1	1.2

*for *M* > 800000 *a* = 0.55.

**Table 2:** Measured intrinsic viscosities for HA:TSP mixtures compared with the theoretically predicted (based on values from Table 1) if there was no interaction.

HA:TSP ratio	[η]	[η] (theoretical)
1:3 1:1 3:1	780 mL/g 1100 mL/g 1180 mL/g	940 mL/g 1075 mL/g 1200 mL/g
